# Phase I study of single agent NIZ985, a recombinant heterodimeric IL-15 agonist, in adult patients with metastatic or unresectable solid tumors

**DOI:** 10.1136/jitc-2021-003388

**Published:** 2021-11-19

**Authors:** Kevin Conlon, Dionysios C Watson, Thomas A Waldmann, Antonio Valentin, Cristina Bergamaschi, Barbara K Felber, Cody J Peer, William D Figg, E Lake Potter, Mario Roederer, Douglas G McNeel, John A Thompson, Sumati Gupta, Rom Leidner, Andrea Wang-Gillam, Nehal S Parikh, Debby Long, Sema Kurtulus, Lang Ho Lee, Niladri Roy Chowdhury, Florent Bender, George N Pavlakis

**Affiliations:** 1 Lymphoid Malignancies Branch, Center for Cancer Research, National Cancer Institute, Bethesda, Maryland, USA; 2 Human Retrovirus Section, Vaccine Branch, Center for Cancer Research, National Cancer Institute at Frederick, Frederick, Maryland, USA; 3 University Hospitals Cleveland Medical Center, Cleveland, Ohio, USA; 4 Human Retrovirus Pathogenesis Section, Vaccine Branch, Center for Cancer Research, National Cancer Institute, Frederick, Maryland, USA; 5 Clinical Pharmacology Program, Center for Cancer Research, NCI, Bethesda, Maryland, USA; 6 Vaccine Research Center, NIAID, Bethesda, Maryland, USA; 7 Carbone Cancer Center, University of Wisconsin Madison, Madison, Wisconsin, USA; 8 Seattle Cancer Care Alliance, Seattle, Washington, USA; 9 Huntsman Cancer Institute, University of Utah, Salt Lake City, Utah, USA; 10 Earle A Chiles Research Institute, Providence Cancer Institute, Portland, Oregon, USA; 11 Division of Oncology, Department of Medicine, Washington University in Saint Louis, St Louis, Missouri, USA; 12 Novartis Institutes for BioMedical Research Inc, Cambridge, Massachusetts, USA

**Keywords:** clinical trials as topic, therapies, investigational, cytokines, immunotherapy, immunomodulation

## Abstract

**Background:**

NIZ985 is a recombinant heterodimer of physiologically active interleukin (IL-)15 and IL-15 receptor alpha. In preclinical models, NIZ985 promotes cytotoxic lymphocyte proliferation, killing function, and organ/tumor infiltration, with resultant anticancer effects. In this first-in-human study, we assessed the safety, pharmacokinetics, and immune effects of NIZ985 in patients with metastatic or unresectable solid tumors.

**Methods:**

Single agent NIZ985 dose escalation data are reported from a phase I dose escalation/expansion study of NIZ985 as monotherapy. Adult patients (N=14) received 0.25, 0.5, 1, 2 or 4 µg/kg subcutaneous NIZ985 three times weekly (TIW) for the first 2 weeks of each 28-day cycle, in an accelerated 3+3 dose escalation trial design. IL-15 and endogenous cytokines were monitored by ELISA and multiplexed electrochemiluminescent assays. Multiparameter flow cytometry assessed the frequency, phenotype and proliferation of peripheral blood mononuclear cells. Preliminary antitumor activity was assessed by overall response rate (Response Evaluation Criteria in Solid Tumors V.1.1).

**Results:**

As of March 2, 2020, median treatment duration was 7.5 weeks (range 1.1–77.1). Thirteen patients had discontinued and one (uveal melanoma) remains on treatment with stable disease. Best clinical response was stable disease (3 of 14 patients; 21%). The most frequent adverse events (AEs) were circular erythematous injection site reactions (100%), chills (71%), fatigue (57%), and fever (50%). Treatment-related grade 3/4 AEs occurred in six participants (43%); treatment-related serious AEs (SAEs) in three (21%). The per-protocol maximum tolerated dose was not reached. Pharmacokinetic accumulation of serum IL-15 in the first week was followed by significantly lower levels in week 2, likely due to more rapid cytokine consumption by an expanding lymphocyte pool. NIZ985 treatment was associated with increases in several cytokines, including interferon (IFN)-γ, IL-18, C-X-C motif chemokine ligand 10, and tumor necrosis factor-β, plus significant induction of cytotoxic lymphocyte proliferation (including natural killer and CD8^+^ T cells), increased CD16^+^ monocytes, and increased CD163^+^ macrophages at injection sites.

**Conclusions:**

Subcutaneous NIZ985 TIW was generally well tolerated in patients with advanced cancer and produced immune activation paralleling preclinical observations, with induction of IFN-γ and proliferation of cytotoxic lymphocytes. Due to delayed SAEs at the two highest dose levels, administration is being changed to once-weekly in a revised protocol, as monotherapy and combined with checkpoint inhibitor spartalizumab. These alterations are expected to maximize the potential of NIZ985 as a novel immunotherapy.

**Trial registration number:**

NCT02452268.

## Introduction

Interleukin (IL)-15 is a common γ chain cytokine with a distinctive role in the development, survival, proliferation, and activation of lymphocytes, including natural killer (NK) cells, canonical αβTCR^+^CD8^+^, γδTCR^+^, NKT, and intraepithelial T lymphocytes.^1^ IL-15 shares a common gamma chain receptor with IL-2, the prototypical immunotherapeutic oncology agent,^2^ and has similar biological effects. However, key differences in production, receptor affinity and secretion confer unique properties to IL-15, and advantages over IL-2 for the treatment of cancer. Specifically, endogenous IL-2 stimulates T cell activity but subsequently protects against the risk of autoimmunity by eliminating self-reactive T cells through a combination of T-regulatory (Treg) cell maintenance and the promotion of activation-induced cell death (AICD), particularly at low cytokine concentrations.^2–4^ On the other hand, high-dose IL-2 therapy is associated with severe toxicities including hypotension, renal failure and capillary leak syndrome.^5^ These detrimental factors have suggested a need for cytokine therapies possessing the immunostimulatory effects of IL-2, but with fewer adverse effects.

In contrast to IL-2, IL-15 has been shown to inhibit IL-2 mediated AICD in transgenic mice^6^ and not lead to Treg expansion,^7^ while maintaining the ability to augment cytotoxic immune response. Given the more favorable immunologic profile of IL-15, we and others have explored its potential as an immunostimulatory cytokine for the treatment of cancer.^8^

IL-15 is co-expressed with an IL-15 binding protein, IL-15 receptor alpha (IL-15Rα), a membrane anchored, stabilizing polypeptide crucial to the natural biosynthesis of the cytokine.^9–12^ The two polypeptide chains form a complex in the endoplasmic reticulum, and undergo glycosylation and traffic through the Golgi to the plasma membrane.^13–15^ The membrane-anchored IL-15Rα is responsible for IL-15 retention on the cell surface, where it is trans-presented to adjacent responding cells expressing the IL-2/IL-15 βγ receptor.^16^ In addition, after a specific proteolytic cleavage of IL-15Rα,^17^ a soluble heterodimeric form of IL-15:IL-15Rα is released, circulates in the blood, and is biologically active.^13 17 18^ Thus, although IL-15Rα was hypothesized to be part of the IL-2/IL-15 receptor βγ complex by analogy to IL-2Rα, it is instead part of the physiological cytokine, produced as heterodimeric IL-15, either cell-associated or soluble.

To leverage the advantageous features of IL-15, we produced a recombinant heterodimeric IL-15 molecule (hetIL-15) with high homology to the naturally produced IL-15 that circulates in human plasma.^17^ Administration of the recombinant heterodimer produced in human cells has been well tolerated and bioactive across a wide range of doses in mice and macaques, leading to expansion and tissue trafficking of cytotoxic lymphocytes.^17 19–21^ While having similar in vitro effects to recombinant single-chain monomeric IL-15 produced in bacteria, the heterodimer presents significant advantages in vivo, with several studies in mice and macaques showing superior pharmacokinetics (PK) and a 10-fold to 100-fold increase in agonistic activity over single-chain IL-15.^13 17 20 22–24^

Extensive studies in murine preclinical models have demonstrated the efficacy of monomeric or heterodimeric IL-15 to treat cancers. IL-15 enhanced the in vivo antitumor activity of tumor-reactive CD8^+^ T cells^25 26^ and prolonged the survival of mice in several tumor models including colon carcinoma, prostatic cancer, melanoma, breast, myeloma and pancreatic models.^22 24 27–33^ Recent studies have investigated the mechanism by which IL-15 induces lymphocyte entry into tumors and increases their cytotoxicity.^27^

Different IL-15 formulations have been tested in several cancer clinical trials. Single-chain recombinant IL-15 has been tested in humans as intravenous bolus (30 min infusion),^34 35^ subcutaneous (SC) injection^35 36^ and continuous intravenous infusion.^37^ In these studies, similar to preclinical models, IL-15 was shown to affect lymphocyte homeostasis through both redistribution and proliferation of target cells. However, the administration of recombinant single-chain IL-15 as a bolus resulted in significant toxicities including fever, chills and blood pressure changes occurring 2–4 hours after treatment. Pharmacokinetics revealed that serum concentrations of IL-15 peaked in a similar time frame, and subsequently dropped in a rapid fashion. In contrast, another clinically tested formulation of IL-15 in association with IL-15Rα fused to the Fc domain of immunoglobulin G (ALT-803) showed more prolonged cytokine serum levels and appeared to be better tolerated.^38 39^ ALT-803 has also been tested as an intravenous infusion and SC injection in patients with advanced solid tumors^38^ or with relapsed hematologic malignancy following allogeneic hematopoietic stem cell transplantation.^39^

In this study, we report on the safety and preliminary activity of the recombinant, heterodimeric form of IL-15:IL-15Rα (NIZ985/hetIL-15) in patients with metastatic or unresectable solid tumors in a phase I first-in-human dose escalating study of NIZ985 as a single agent.

## Materials and methods

### Clinical study design and objectives

This is an open-label, phase I dose escalation/expansion study initially evaluating the safety and efficacy of NIZ985 administered by SC injection three times weekly (TIW) as a single agent for two consecutive weeks of a 4-week treatment cycle, in patients with metastatic or unresectable cancers. Subsequent protocol amendments introduced assessments of combination therapy with the anti-programmed cell death protein 1 (PD-1) monoclonal antibody spartalizumab, and an alternative once-weekly NIZ985 administration schedule. Herein are reported data from the original NIZ985 single agent TIW dose escalation design.

Dose escalation initially followed an accelerated design with single patient cohorts until a grade 2 adverse event (AE) occurred, when cohort size was switched to the standard 3+3 patient phase I design. Treatment was continued in the absence of dose-limiting toxicity (DLT) or other unacceptable AE, disease progression (assessed radiographically every other cycle), or patient withdrawal. Patient enrollment commenced in July 2015 and 14 patients were treated at a total of five clinical sites.

The primary objective of the study was to assess the safety, toxicity profile and DLT, and determine the maximum tolerated dose of SC NIZ985 administered TIW. Secondary objectives were to determine the PK profile of NIZ985; to characterize its pharmacodynamic effects on the percentages and absolute numbers of circulating lymphocyte subsets; to measure changes in circulating levels of cytokines; and undertake a preliminary evaluation of antitumor activity by determining the overall response rate by Response Evaluation Criteria in Solid Tumors (RECIST) V.1.1 criteria, time to disease progression, progression-free survival, and overall survival.

## Patient selection

Eligible patients were adults at least 18 years of age, with histologically confirmed metastatic or unresectable solid tumor malignancy and progression on at least one prior therapy, for whom curative or palliative measures were non-existent or associated with minimal survival benefit. Evaluable or measurable disease was required, defined as at least one lesion accurately measurable in at least one dimension as being ≥20 mm by conventional techniques or ≥10 mm by spiral CT. Patients required an Eastern Cooperative Oncology Group performance status of 0 or 1, and pulmonary function tests (diffusing capacity/alveolar volume and forced expiratory volume) at least 50% of predicted. Patients also required normal organ and marrow function, defined as: (1) leukocyte, absolute neutrophil, and platelet counts per µL of at least 3000, 1500, and 100,000 cells, respectively; (2) alanine and aspartate aminotransferases no more than 2.5 × the upper limit of normal (ULN); (3) a total bilirubin level within normal limits; and (4) either serum creatinine <1.5 × ULN or serum creatinine ≥1.5 × ULN with creatinine clearance ≥60 mL/min/1.75 m^2^.

Patients could not have primary brain cancers or active central nervous system metastases, or other types of malignant disease other than those treated in the study, with the exception of prior malignancies that had been curatively treated without recurrence for at least 2 years or completely resected. Furthermore, active known or suspected autoimmune conditions were excluded, with the exception of those requiring an external trigger, adequately managed conditions associated with prior anti-PD-1 or anti-programmed cell death ligand 1 (PD-L1) treatment, or non-relevant conditions such as vitiligo or type 1 diabetes. Other intercurrent illnesses that would exclude patients included clinically significant congestive heart disease and arrhythmias, severe asthma or an absolute requirement for oral corticosteroids (earlier versions of the protocol also excluded inhalant corticosteroids), active bacterial infections, documented HIV infection or a positive serology for HIV disease, and either serologic or PCR evidence of hepatitis B or C infection. Pregnant female patients were excluded. Prior IL-15 treatment was an exclusion criterion, as was any concurrent anticancer therapy apart from bisphosphonates, or hormone therapy for metastatic prostate cancer which had progressed despite castrate levels of testosterone. Other previous treatments must have ended more than 4 weeks prior to enrollment—or more than 6 weeks before first study dose in the case of prior checkpoint inhibitors, nitrosoureas, or mitomycin C—and patients must have recovered from prior treatment toxicities (Common Terminology Criteria for Adverse Events grade 0 or 1).

### Treatment plan and drug administration

NIZ985 was administered SC on Monday, Wednesday and Friday for the first 2 weeks of 28-day treatment cycles at doses of 0.25, 0.5, 1.0, 2.0 or 4.0 µg/kg. Treatment was continued in the absence of unacceptable toxicity, progressive disease per RECIST V.1.1, patient or treating physician’s decision to discontinue treatment, or the patient’s death. While no patient experienced a DLT, three patients at the two highest dose levels of 2 and 4 µg/kg experienced serious AEs during cycle 2 of therapy (discussed in safety results, below), prompting a halt in the dose escalation and the enrollment of three new patients at 1 µg/kg to confirm this as a safe and tolerable level for TIW dosing. The first nine patients enrolled (dose levels 0.25 µg/kg through 2 µg/kg, including the first three dosed at 1 µg/kg) were treated at a single center at the National Cancer Institute, and the remaining five patients (two at 4 µg/kg and an additional three at 1 µg/kg) were enrolled at the other four sites. All patients received concomitant medication consisting of antipyretics, acetaminophen or non-steroidal anti-inflammatory drugs, anti-emetics, antihistamines and anti-diarrheals either as premedication or on an as-needed basis as indicated by their tolerance of the treatment, as well as other supportive medications, including intravenous fluids and electrolyte or mineral replacement to address any AEs to improve their toleration of the treatment.

### Safety and response assessments

Patients were monitored during treatment with regular scheduled physical and laboratory examinations to aid in the assessment of their condition while receiving the study medications. Patients were seen on each of their treatment days and once weekly during weeks 3 and 4 of cycle 1 for physical examination and evaluation of injection site reactions, and had regular chemistry and hematology panels.

### Pharmacokinetic, pharmacodynamic, and biomarker analysis

To characterize the PK parameters of NIZ985, blood samples were collected from all patients at pre-dose, throughout the study, and at the end of treatment. NIZ985 serum concentrations were measured using a good laboratory practice (GLP)-validated electrochemiluminescent (ECL) immunoassay specific for the NIZ985 heterodimer, with a lower limit of quantitation (LLOQ) of 50 pg/mL in 100% human serum. NIZ985 PK parameters were derived from concentrations determined with the GLP assay at recorded sampling times using non-compartmental methods in Phoenix WinNonlin V.6.2 (Certara USA, Princeton, New Jersey, USA). Derived parameters included the maximum serum concentration (C_max_), time to C_max_ (T_max_), time to last evaluable measurement (T_last_), concentration at T_last_ (C_last_), and the area under the concentration–time curve from 0 to T_last_ (AUC_last_).

Circulating IL-15 was also evaluated using a fit-for-purpose Meso Scale Discovery (MSD) human V-plex ECL assay (Meso Scale Diagnostics, Rockville, Maryland, USA) as supportive surrogate data for NIZ985^18^ in a post-hoc exploratory analysis of PK in the first nine patients treated. This assay, which is reported to recognize IL-15 both as a single-chain molecule and in association with IL-15Rα, has a lower limit of detection of 1.4 pg/mL and was performed according to the manufacturer’s instructions; data were reported as equivalent single chain IL-15.

Systemic cytokines and other biomarkers were measured pretreatment and post-treatment in serum samples using the MSD V-PLEX Human Cytokine 30-plex and MSD V-Plex Human Biomarker 54-plex kits, according to manufacturer’s instructions.

Patients had flow cytometry assessments for lymphocyte and monocyte cell subsets at baseline and while on-treatment to follow immune cell population changes. For the last five patients treated (after the study became multicenter) flow analyses were performed using the methodology and antibody panel described in online supplemental table S1. For the first nine patients treated, flow cytometry was performed using cryopreserved longitudinal samples of peripheral blood mononuclear cells (PBMC). Frozen PBMC were thawed using Thawsome adaptors^40^ into 9 mL warmed R10, washed with phosphate-buffered saline, and incubated with 100 µL of a 1:800 dilution of the viability dye UV Blue (Invitrogen) for 20 min. After incubation, the cells were washed once with staining buffer (RPMI 1680 supplemented with 4% heat-inactivated fetal calf serum) and incubated with the extracellular antibody cocktails, made with 20× Super Bright Staining Buffer (eBioscience), for 20 min at room temperature. After the incubation, the cells were washed with staining buffer and permeabilized with FoxP3 Fixation/Permeabilization buffer (eBioscience) at room temperature for 30 min. Cells were washed with 1× Permeabilization Buffer (eBioscience) and incubated with 100 µL of the intracellular antibody cocktail, made in 1× perm buffer, at room temperature for 30 min. Cells were washed with 1× perm buffer and resuspended in phosphate-buffered saline. Samples were run on a BD FACSymphony flow cytometer. Data were analyzed using the FlowJo software platform. Antibody panel details are given in online supplemental table S2.

10.1136/jitc-2021-003388.supp1 Supplementary data

## Results

### Patient population and treatment

As of March 2, 2020, a total of 14 adult patients with advanced metastatic cancers were treated in an accelerated dose escalation design with NIZ985 doses of 0.25 µg/kg (n=1), 0.5 µg/kg (n=2), 1.0 µg/kg (n=6), 2.0 µg/kg (n=3) and 4.0 µg/kg (n=2). Baseline characteristics are shown in table 1. Study participants were heavily pretreated, with 50% having received three or more prior treatments and nearly two-thirds prior immunotherapy for a variety of solid tumors, of which only a minority (3/14) were classical immunosensitive diagnoses of renal cell carcinoma or melanoma (online supplemental table S3). By data cut-off (March 2, 2020) 13 participants had discontinued, primarily for progressive disease (online supplemental table S4). The median duration of treatment was two cycles, with a range between <1 (three doses; patient discontinued for a treatment-unrelated AE) to 19 cycles (online supplemental figure S1).

**Table 1:** Participant demographics

Characteristic	NIZ985 0.25 µg/kg n=1	NIZ985 0.5 µg/kg n=2	NIZ985 1.0 µg/kg n=6	NIZ985 2.0 µg/kg n=3	NIZ985 4.0 µg/kg n=2	All NIZ985 patients N=14
Median age, years (range)	50 (NA)	62 (59–64)	53 (42–69)	53 (52–73)	63 (58–67)	57 (42–73)
Age category, n (%)						
<50 years	0	0	2 (33.3)	0	0	2 (14.3)
50 to <65 years	1 (100)	2 (100)	3 (50.0)	2 (66.7)	1 (50.0)	9 (64.3)
≥65 years	0	0	1 (16.7)	1 (33.3)	1 (50.0)	3 (21.4)
Sex, n (%)						
Male	1 (100)	1 (50.0)	4 (66.7)	1 (33.3)	1 (50.0)	8 (57.1)
Female	0	1 (50.0)	2 (33.3)	2 (66.7)	1 (50.0)	6 (42.9)
Race, n (%)						
Black/African American	0	0	1 (16.7)	0	0	1 (7.1)
White	1 (100)	2 (100)	5 (83.3)	2 (66.7)	2 (100)	12 (85.7)
Unknown	0	0	0	1 (33.3)	0	1 (7.1)
Ethnicity, n (%)						
Hispanic/Latino	0	0	1 (16.7)	1 (33.3)	0	2 (14.3)
Other	1 (100)	2 (100)	5 (83.3)	2 (66.7)	1 (50.0)	11 (78.6)
Not reported	0	0	0	0	1 (50.0)	1 (7.1)

### Safety

**Figure 1:**
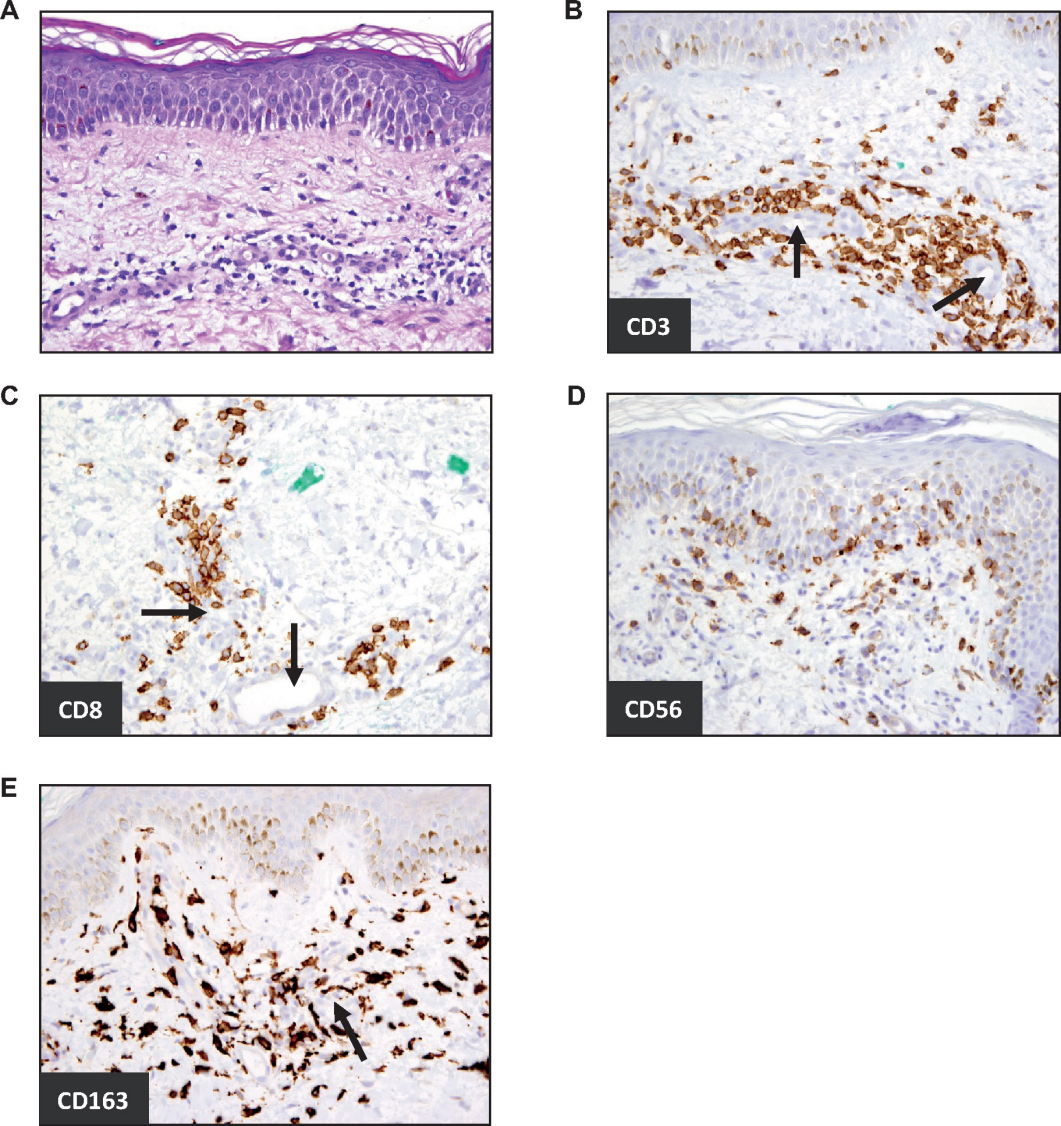
NIZ985 injection site reaction is characterized by local immune cell infiltration. Micrographs from skin biopsy of patient treated with 2 μg/kg NIZ985. (A) H&E stain demonstrated subacute spongiotic dermatitis with superficial perivascular inflammation. (B–E) Immunohistochemistry micrographs for immune cell markers. (B) CD3^+^ cells (T cells) and (C) CD8^+^ cells were primarily found near dermal blood vessels (black arrows). (D) CD56^+^ cells were also found near the dermal/epidermal junction, while (E) CD163^+^ cells (monocyte lineage) were prominent throughout the affected dermis.

NIZ985 treatment was generally well tolerated, particularly at the initial dose levels. Table 2 summarizes treatment-related AEs and serious AEs (SAEs) regardless of study drug relationship, and online supplemental table S5 summarizes all AEs. Injection site reactions (see below), chills, fatigue, and fever were the most common treatment-related events, along with characteristic cytokine-related events like anorexia, followed by arthralgias, hyperhidrosis and gastrointestinal events that occurred in about 25% of the patients. There was no clear relationship between the frequency or severity of these AEs and the dose level. Grade 3 or 4 AEs occurred in six patients overall at rates of approximately 30%–50% in the three intermediate dose levels (0.5–2 µg/kg) and in both of the 4 µg/kg patients (online supplemental table S5). Treatment-related grade 3 or 4 AEs were dissimilar among these six patients (table 2), and most unrelated grade 3 or 4 AEs were attributable to tumor pain or chronic organ/system dysfunction due to underlying disease or prior treatment. With the exception of one patient, the circular, erythema multiforme-like injection site reactions (ISRs) were not problematic in terms of pain, edema or size. ISRs increased with increasing dose, and patients treated at higher doses developed more notably visible circular areas of erythema and mild tenderness beginning at the site of their treatment injection within 24–48 hours of injection. These continued to enlarge circumferentially and occasionally reached 30 cm in diameter, but most often did not require analgesics. Clearance occurred circumferentially from the center, often resulting in a bullseye appearance of erythema (online supplemental figure S2). Of note, ISR weal size was observed to diminish with each subsequent cycle. Pathologic and immunohistochemical analyses of these injection site reactions showed perivascular infiltrate of CD3^+^ CD4^+^ lymphocytes, and CD56^+^ lymphocyte infiltration at the dermal–epidermal junction as well as increased number of CD3^+^CD8^+^ lymphocytes and monocyte/macrophages in the tissue (figure 1).

**Table 2:** Treatment-related adverse events and serious adverse events

	NIZ985 0.25 µg/kg n=1	NIZ985 0.5 µg/kg n=2	NIZ985 1.0 µg/kg n=6	NIZ985 2.0 µg/kg n=3	NIZ985 4.0 µg/kg n=2	All NIZ98 patients N=14
Treatment-related events						
Any event	1 (100)	2 (100)	6 (100)	3 (100)	2 (100)	14 (100)
Grade 3–4 events	0	1 (50.0)*	2 (33.3)†	1 (33.3)‡	2 (100)§	6 (42.9)
Common treatment-related events (>20% in all patients)						
ISR	1 (100)	2 (100)	6 (100)	3 (100)	2 (100)	14 (100)
Chills	0	1 (50.0)	4 (66.7)	3 (100)	2 (100)	10 (71.4)
Fatigue	0	1 (50.0)	4 (66.7)	3 (100)	0	8 (57.1)
Fever	0	1 (50.0)	4 (66.7)	2 (66.7)	0	7 (50.0)
Arthralgia	0	2 (100)	2 (33.3)	1 (33.3)	0	5 (35.7)
Nausea	0	1 (50.0)	2 (33.3)	1 (33.3)	1 (50.0)	5 (35.7)
Hyperhidrosis	0	0	2 (33.3)	2 (66.7)	0	4 (28.6)
Decreased appetite	0	0	2 (33.3)	1 (33.3)	0	3 (21.4)
Constipation	0	0	1 (16.7)	2 (66.7)	0	3 (21.4)
Cognitive disorder	0	1 (50.0)	1 (16.7)	0	1 (50.0)	3 (21.4)
Serious adverse events						
Any SAE	0	0	0	2 (66.7)	1 (50.0)	3 (21.4)
Acute kidney Injury	0	0	0	0	1 (50.0)	1 (7.1)
Oliguria	0	0	0	0	1 (50.0)	1 (7.1)
Dermatitis bullous	0	0	0	1 (33.3)	0	1 (7.1)
Purpura	0	0	0	1 (33.3)	0	1 (7.1)
Embolism	0	0	0	0	1 (50.0)	1 (7.1)
Vasculitis	0	0	0	0	1 (50.0)	1 (7.1)

*Grade 3 anemia.

†Grade 3 ISR (misclassified; subsequently reviewed and reclassified as grade 2) and grade 3 diarrhea.

‡Grade 3 increase in international normalized ratio.

§Grade 3 hyponatremia (one patient); grade 3 lymphocyte count decreases (two events), peripheral edema, purpura, and oliguria, plus grade 3 and 4 events of embolism, acute kidney injury, and vasculitis (one patient).

ISR, injection site reaction; SAE, serious adverse event.

There were three medically significant SAEs (table 2) that occurred during the second cycle of treatment in patients receiving 2 and 4 µg/kg. The first 2 µg/kg patient developed new diffuse vesicles soon after the day 12 injection that subsequently progressed to widespread multiple painful bullae. The patient began treatment with systemic corticosteroids and required 40 days of treatment for resolution of lesions. Pathologic examination confirmed the diagnosis of immunoglobulin A (IgA) bullous dermatitis or pemphigoid. The second 2 µg/kg patient developed purpuric lesions largely on the lower extremities during the last week of cycle 2 associated with fatigue, fevers and arthralgias, with the purpura caused by supratherapeutic INR in the setting of warfarin treatment for prior pulmonary embolus. Biopsy showed a mild perivascular lymphocytic infiltrate with no vasculitis, and the event was ultimately attributed to excess anticoagulation, which resolved after interruption of warfarin and administration of fresh frozen plasma and vitamin K. The third SAE occurred in a 4 µg/kg patient who presented with symptoms of dehydration and tachycardia following the fourth dose of cycle 2. A CT pulmonary angiogram showed bilateral pulmonary emboli and the patient was admitted to hospital for anticoagulation and clinical management. On the second day of admission, the patient developed lower extremity purpuric lesions. Immunofluorescent biopsy of the left thigh and left forearm demonstrated IgA vasculitis, and the patient received 8 days of prednisone 40 mg/day before tapering off corticosteroid treatment. The patient further developed non-obstructive acute kidney injury requiring a dialysis treatment. The patient’s condition improved, however they were lost to follow-up following transition to hospice care due to disease progression.

### Preliminary efficacy

There were no complete or partial responses observed, and the best response of stable disease (SD) in three participants (21%) did not appear to be dose-dependent (online supplemental figure S3). Nine patients discontinued for confirmed disease progression and three for AEs, one of which (small bowel obstruction) was determined to be due to disease progression during the first week of treatment. As seen in online supplemental figure S3, three participants had dramatic enlargement of their tumor burden and the remaining 10 with available data had mostly minor changes in their target lesions. Of the three participants achieving SD, one (metastatic renal cell carcinoma) had an SD duration of 28 days, one (cholangiocarcinoma) withdrew consent after 1 day of SD, and, of note, one has had prolonged disease stabilization for over 15 months at cut-off with metastatic uveal melanoma, a diagnosis normally associated with variable response to therapy and a poor outcome. This patient remains on treatment at time of writing.

### Pharmacokinetic data

**Figure 2:**
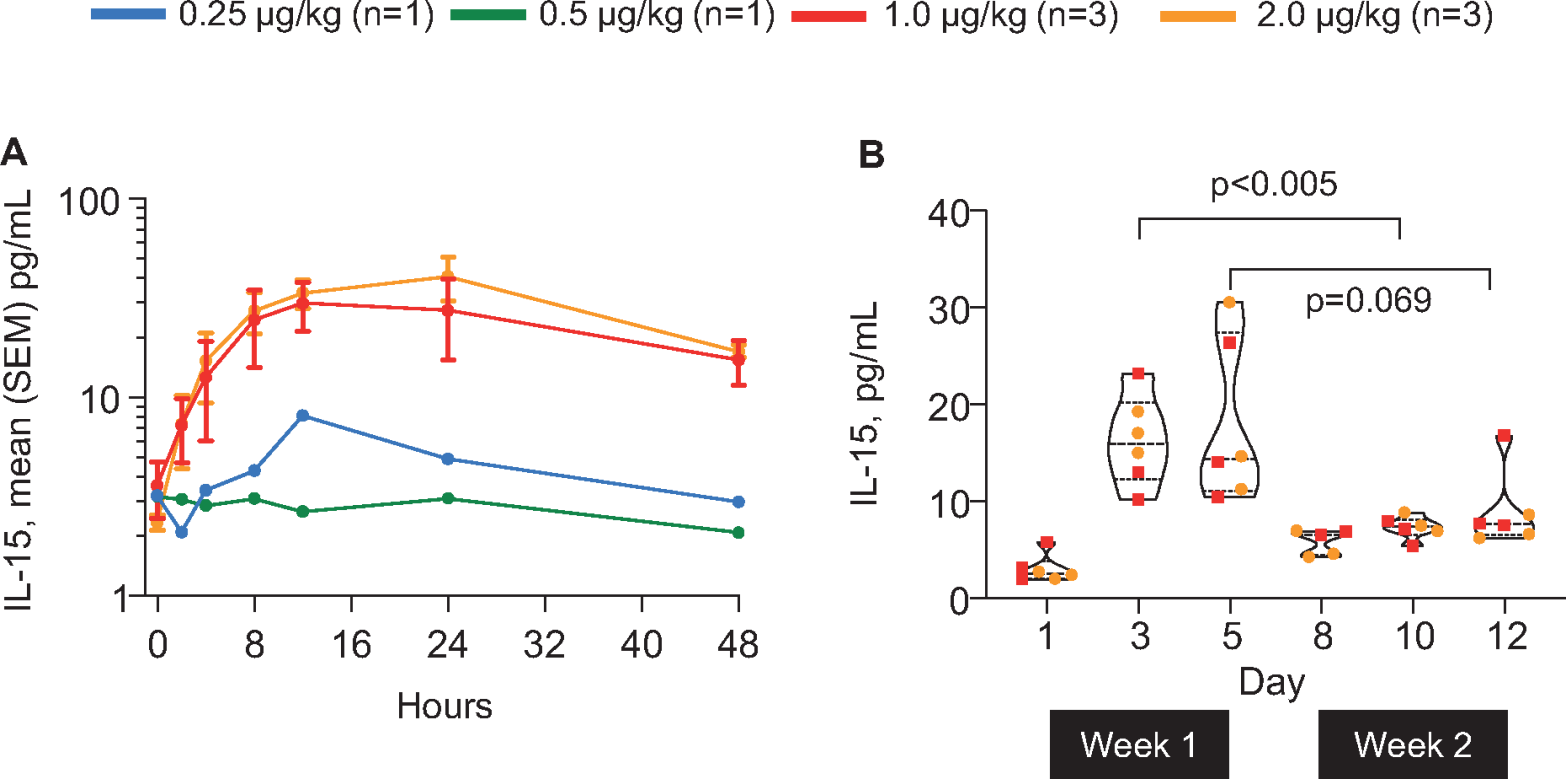
Pharmacokinetics and circulating serum IL-15 concentrations during cycle 1 of NIZ985 treatment in a subset of participants. (A) Serum IL-15 concentration–time profile (mean±SEM) after subcutaneous injection of NIZ985 on cycle 1, day 1. (B) Pooled analysis of serum IL-15 levels immediately before each injection of NIZ985 (1 and 2 μg/kg groups). Violin plots depict median, quartiles and range of data. Each data point represents an individual patient sample. Comparison between indicated time points was by mixed-effects ANOVA. ANOVA, analysis of variance; IL, interleukin.

Due to a high number of samples below the NIZ985 heterodimer-specific ECL assay LLOQ, consistent concentration–time data and derived PK parameters were only available for five patients in the 1–4 µg/kg dosing groups (table 3). T_max_ was highly variable (7–27 hours) and both C_max_ and AUC_last_ appeared similar among patients receiving 1 or 2 µg/kg but were higher for the patient administered 4 µg/kg. Due to the limited number of evaluable PK samples and low numbers of patients, dose proportionality could not be assessed. Supportive post-hoc analyses of serum IL-15 in the first eight patients treated at doses between 0.25 and 2 µg/kg, using the Mesoscale ECL assay with a lower detection limit, also showed comparable concentration–time profiles at cycle 1, day 1 for patients receiving 1 or 2 µg/kg (figure 2A), as well as a suggestion of accumulation over the first week of cycle 1 treatment followed by significantly lower exposures in the second week (figure 2B).

**Table 3:** NIZ985 pharmacokinetic parameters

Participant	Dose (µg/kg)	C_max_ (pg/mL)	T_max_ (hr)	AUC_last_ (pg·h/mL)	C_last_ (pg/mL)	T_last_ (h)
1	1	132	24.0	4085	52.6	44.4
2	1	120	27.4	2838	120	27.4
3	2	185	23.0	5126	56.1	46.1
4	2	138	12.0	3975	60.3	43.0
5	4	461	7.17	10 910	70.3	49.1

NIZ985 concentrations in the 0.25 ug/kg and 0.5 ug/kg TIW cohorts were below the assay LLOQ (50 pg/mL). In these two cohorts, samples were analyzed beyond the current demonstrated stability period of 710 days. Hence, measurements from these cohorts were not reported.

AUC_last_, area under the NIZ985 concentration–time curve from 0 to T_last_; C_last_, last quantifiable NIZ985 serum concentration; C_max_, maximum NIZ985 serum concentration; T_last_, time post-dose of C_last_; T_max_, time post-dose of C_max_.

### Immunological and biomarker changes in peripheral blood

#### IL-15 effects on blood leukocytes

**Figure 3:**
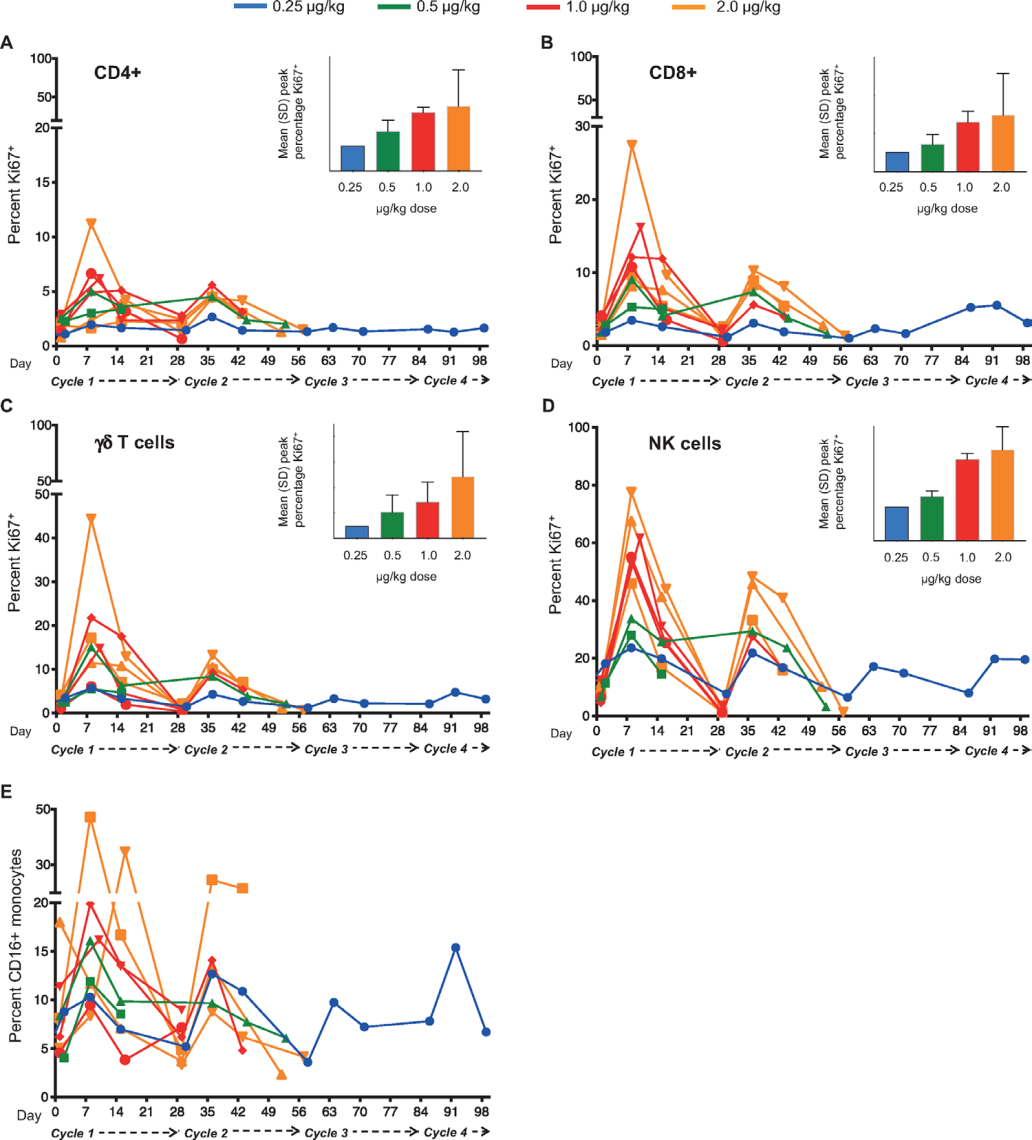
Lymphocyte proliferation is consistently induced during NIZ985 treatment cycles. (A–D) Individual proliferative responses (percentage Ki67^+^ cells) induced during each cycle of NIZ985 in circulating CD4^+^ T cells, CD8^+^ T cells, γδ T cells, and NK cells. Insets show mean peak percentage Ki67^+^ cells by dosing group; (E) Individual percentage CD16^+^ monocytes (from total CD14^+^ monocytes) during each cycle of NIZ985. NK, natural killer.

Among the first nine patients treated, dose-related increases in the expression of Ki67 were observed in several leukocyte subsets—including CD4^+^ and CD8^+^αβ T lymphocytes, γδ T lymphocytes and NK cells—over the course of the first 2–3 cycles (figure 3A–D). Similar increases were observed among the remaining five patients analyzed separately (online supplemental figure S4) using the methodology and antibody panel described in online supplemental table S1. Doses of 0.25 or 0.5 µg/kg NIZ985 evidenced modest post-baseline increases in Ki67^+^ cell percentages, whereas higher doses of 1 and 2 µg/kg were associated with greater mean increases in Ki67^+^ NK cells (data summarized in online supplemental table S6).

We also evaluated the levels of absolute lymphocyte counts after NIZ985 administration. The most consistent increase in absolute blood counts was in NK cells, which increased by 1.2-fold to 4.8-fold in different patients (online supplemental figure S5). Despite high levels of proliferation, we did not observe a consistent increase in the absolute numbers of circulating CD8^+^ or CD4^+^ T cells (online supplemental figure S5), possibly a result of rapid migration out of the blood and into effector sites (see also Discussion). Consistent with the lack of IL-15 receptor expression, no significant changes were observed in the frequency or proliferative responses of B lymphocytes (not shown). Interestingly, although monocytes also do not express the βγ IL-15 receptor, a substantial increase in the frequency of circulating CD16^+^ monocytes was observed in all the analyzed patients (figure 3E). These data suggest that this effect is indirect, probably through the induction of chemokines able to mobilize this monocyte subset, which would be in agreement with data obtained in preclinical animal models.^27^

#### Cytokines

**Figure 4:**
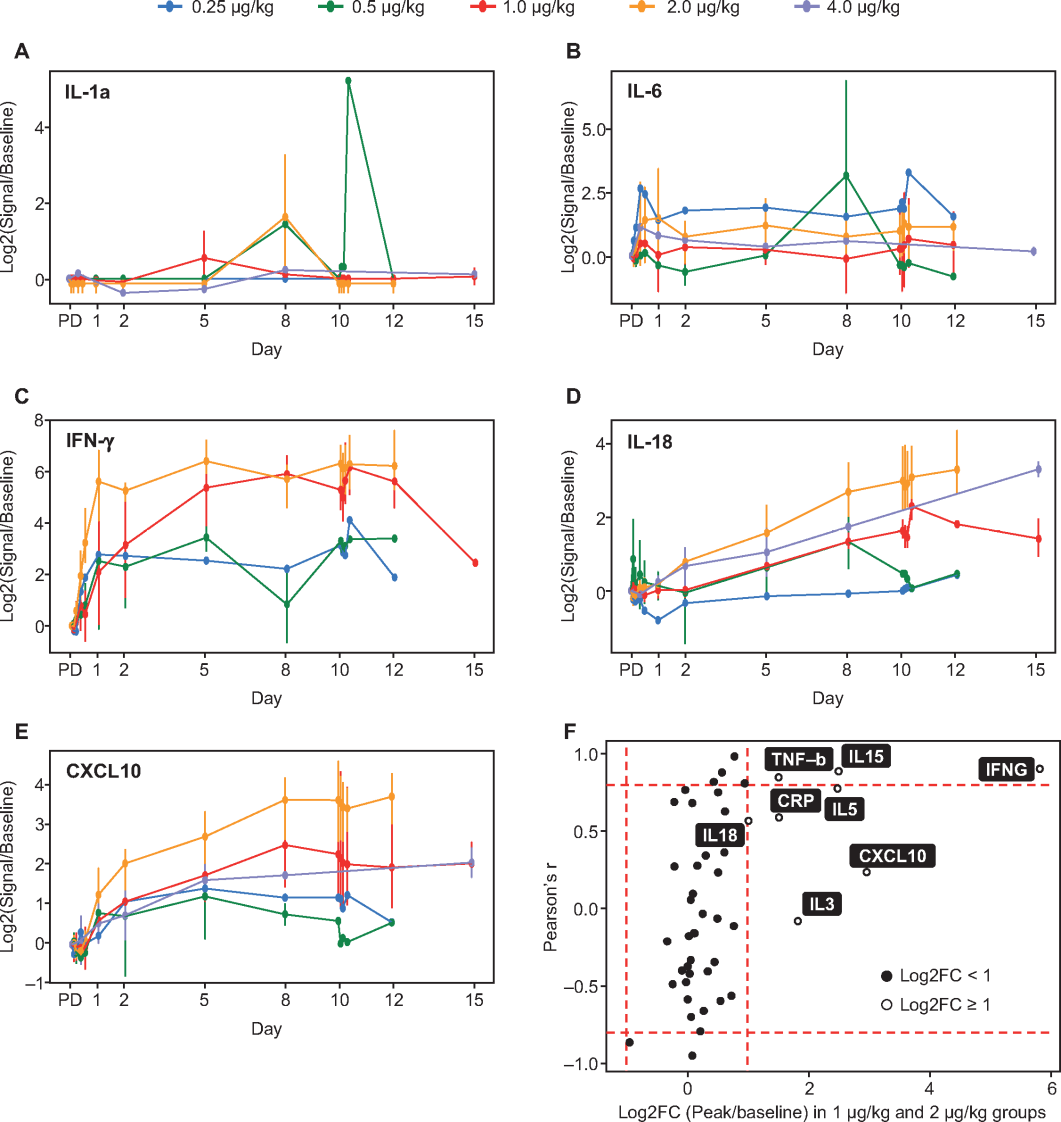
(A–E) Mean peak induction (log2-transformed mean ratios of peak-to-baseline levels) of selected plasma cytokines during cycle 1 dosing; (F) Mean cytokine induction (24–240 hours post-dose) in the pooled 1.0 and 2.0 µg/kg dosing groups. X-axis: Log2-transformed mean ratios of peak to baseline cytokine levels in both groups; Y-axis: Correlation coefficients of the mean ratio across all dose groups and NIZ985 dose level. Bars identify regions of high correlation (Pearson’s r ≥0.8 or ≤–0.8) and high response (≥2-fold). C, cycle; CRP, C-reactive protein; CXCL10, C-X-C motif chemokine ligand 10; D, day; FC, fold-change; IFN, interferon; IL, interleukin; TNF, tumor necrosis factor.

NIZ985 treatment was associated with strong induction in plasma of several cytokines and chemokines including interferon (IFN)-γ, tumor necrosis factor (TNF)-β, C-reactive protein, C-X-C motif chemokine ligand 10 (CXCL10), and IL-5, IL-27, and IL-18. Interpretation of these data is limited by small sample sizes, with generally only one to three evaluable patients per time point with detectable levels of analyte in each dosing group. With this caveat, however, induction appeared to follow one of two patterns (figure 4A–E), with some cytokine levels showing cyclic peaks and others a monotonic rise towards an eventual plateau. The dose relatedness of induction was variable, with high correlations between induction and dose noted for IFN-γ, TNF-β, and IL-27, and lesser correlation for IL-5, IL-18, C-reactive protein, and CXCL10 (figure 4F). We also noted the induction of several chemokines able to recruit monocytes, including IL-18, macrophage inflammatory protein (MIP)-1β/CCL4, MIP-1α/CCL3, and MIP-3α/CCL20. These chemokines were induced by 2×, 1.5×, 1.5× and 1.4×, respectively.

## Discussion

In this study, we present first-in-human data for NIZ985, a recombinant heterodimeric form of IL-15 and IL-15Rα. Treatment was generally well tolerated in this highly refractory set of patients, and produced consistent induction of effector lymphocytes and a preliminary suggestion of clinical activity. Disease stabilization was achieved by 3 of the 14 participants (metastatic renal carcinoma, uveal melanoma, cholangiocarcinoma), consistent with single-agent data for other IL-15 formulations showing stable disease to be the best observed response in solid tumors.^34 36–38^

Although protocol-defined dose-limiting toxicities were not observed, significant systemic immune-related events occurred during cycle 2 in two patients treated in the 2 µg/kg and 4 µg/kg dosing cohorts. Since both were IgA antibody mediated, their immunopathophysiology is difficult to connect to the known biology of IL-15. Bullous skin disorders characterized by complement and IgG deposition have been previously reported in patients receiving checkpoint inhibitors,^41^ as has IgA nephropathy,^42 43^ suggesting that agents thought to primarily affect cellular immunity can be associated with antibody-related toxicity. The two significant immune-related toxicities observed suggested a less frequent SC injection schedule may be more advantageous than the TIW schedule explored here, and this rationale was supported by preliminary PK data for serum IL-15 in this study suggesting a similar persistence and accumulation of exogenous heterodimer as previously noted in macaques.^20^ Once-weekly administration of NIZ985 is currently under evaluation under a revised study protocol. Other than these SAEs, the safety profile and tolerability of NIZ985 in this trial was good and would be expected to be similar or better on a once-weekly schedule.

NIZ985 AUC and C_max_ were broadly similar at 1 and 2 µg/kg dosing using the validated NIZ985 heterodimer-specific ECL assay, which is likely to represent a combination of assay limitation, interpatient variability, and paucity of available data. Both parameters were higher in the single patient dosed at 4 µg/kg for whom data were available, but further data are required to assess dose dependency and the exposure–response relationship.

Post-hoc evaluation of serum IL-15 showed favorable kinetics, without the high serum levels previously associated with toxicity in clinical trials of single-chain IL-15.^34–37^ Our concentration–time data suggested a potentially slower elimination rate in humans than the ~12 hour half-life previously seen for SC heterodimer administration in macaques.^20^ The significantly lower steady-state levels of serum IL-15 during week two of treatment are consistent with macaque data^20^ and with the previously observed expansion of IL-15 receptor-bearing lymphocytes on initial administration of IL-15:IL-15Rα heterodimer that reduces free serum levels.^17 20 21 26 34^ This in turn implies a marked in vivo expansion of effector cells in tissues that is not easily measured in peripheral blood, probably due to cell extravasation into effector anatomical sites. If focused at tumor deposits, such expansion would suggest a potential for clinical activity for NIZ985 in combination with other therapeutic agents that may surpass single agent activity.

Pharmacodynamic activity was established for NIZ985 in terms of both lymphocyte expansion and cytokine induction. Dose-related, post-baseline increases in the proportion of Ki67^+^ proliferating cells was noted for T cell subsets and NK cells, similar to previous macaque data, accompanied at higher doses (1 or 2 µg/kg) by substantive (≥2-fold) increases in NK cell numbers. The increase in total body lymphocytes is not measured accurately in blood, because hetIL-15—as well as other formulations of IL-15 administration—induces relocation of lymphocytes to the tissues, as shown in macaques and humans.^20 34^ This rapid and massive relocation of lymphocytes to effector sites creates the impression of lymphopenia in peripheral blood. After the termination of dosing, lymphocytes return gradually to the blood; therefore, measurements of lymphocytes after the end of the NIZ985 cycle show an increase. Patients appear to have different rates of expansion and/or return in peripheral blood, which may be the result of patient history. NK cells behave differently; they are found increased in the blood of most patients after 1 week of hetIL-15 treatment, due probably to the rapid mobilization of new NK cells from the bone marrow. Furthermore, a marked dose-related increase in the frequency of peripheral CD16^+^ monocytic cells was observed despite the absence of an IL-15 receptor target on these cells. Induction of a number of circulating cytokines and other biomarkers in plasma was observed in pooled data from the 1 and 2 µg/kg cohorts, with IL-5, TNF-β, and IFN-γ in particular showing high and strongly dose-correlated levels of induction, and IL-18, IL-3, C-reactive protein, and CXCL10 showing high but less dose-correlated induction. Individual cytokines showed either intermittent peaks of induction after dosing or a steady rise after the first dose. Although these cytokine data are limited by small samples, the different patterns of induction suggest the potential involvement of different pharmacodynamic mechanisms.

In addition to these changes in peripheral blood, immunohistochemical analysis showed immune activation at NIZ985 injection sites that consistently led to a self-limited injection site reaction characterized by infiltration of leukocytes expressing CD3, CD8, CD56 or CD163, regardless of the administered dose. The potential for high local concentrations of NIZ985 to recruit T and NK cells represents a promising characteristic for future studies of intratumoral or local administration of this cytokine. Moreover, the observation of monocytic CD163^+^ cells throughout the injection-site dermis, along with the increases noted in peripheral CD16^+^ monocytes, suggests a cross-talk of NIZ985 effects with antigen presenting cells, which warrants further investigation.^27^

Given the general similarity of the observed PK and pharmacodynamic effects of NIZ985 in this clinical study to previous non-human primate data, it is of interest to note that a step-dose regimen of increasing NIZ985 doses in macaques has been shown to result in more stable cytokine exposure and downstream proliferation of cytotoxic lymphocytes during the course of the treatment cycle that was not associated with increased toxicity.^20^ The results of the step-dose regimen in macaques also suggest that tachyphylaxis, a mechanism postulated to explain experimental results showing that repeated administration of IL-15 resulted in decreasing effects on circulating blood lymphocytes, as also suggested in figure 3. However, our results support an alternative explanation for this phenomenon, which explains all the in vivo observations and our preclinical studies. IL-15 is a homeostatic cytokine and it exists in equilibrium with the lymphocytes.^44^ The predictions of tachyphylaxis were not fulfilled on administration of increasing doses of IL-15, as in step-dosing. In many preclinical experiments, we observed continuous expansion of lymphocytes on increasing doses of hetIL-15.^20 45^ This is the prediction of the homeostatic mechanism. These considerations may be important for the development of optimal clinical protocols for the use of this powerful cytokine.

The incrementally decreasing size of ISR weals observed in this study with increasing duration of exposure to NIZ985 is also a feature of interest. Both these observations emphasize the importance of understanding the unique pharmacology of novel immunotherapeutics in order to optimize their clinical use, and suggest that exploration of a step-dose approach in humans may constitute a way to maximize exposure and efficacy while minimizing toxicity. However, the safety and efficacy of such an approach in oncotherapy remains to be tested.

These data therefore demonstrate NIZ985 to be a bioactive human cytokine causing systemic immune changes potentially favorable for the treatment of cancer.^46–48^ RECIST-defined objective responses were not observed in this patient group, similar to clinical testing of other IL-15 formulations across the spectrum of administration routes and schedules.^34 36–38^ In contrast, a trial of ALT-803 for patients with hematologic malignancies who had relapsed after allogeneic stem cell transplant showed an objective response rate of 19%,^39^ suggesting possible synergy of IL-15 formulations with the underlying graft-versus-leukemia capacity in these transplant patients. The disparity between the preclinical efficacy observed for IL-15 and clinical responses obtained to date implies that a deeper understanding of IL-15 pharmacology and activity is necessary for optimal use. The immunologic effects of NIZ985 could potentially augment the anticancer efficacy of other immunotherapeutic options such as cancer vaccines or checkpoint inhibitors, or other modalities like chemotherapeutics or radiotherapy. The potential for treatment synergy between NIZ985 and checkpoint inhibitors is currently under preliminary evaluation with the anti-PD1 antibody spartalizumab under a protocol amendment to the current study. The strong effects of hetIL-15 on dendritic cell accumulation in tumors of preclinical models^27^ suggest the use of hetIL-15 in combinations aiming to induce tumor antigen presentation and immunotherapeutic response. In addition, the known effects of hetIL-15 on metastatic disease suggest its use in efforts to reduce metastatic dissemination and burden.

Different forms of IL-15 and mimetics such as IL-2/anti-IL-2R antibody complexes have been proposed and tested alone and in combination with other agents—checkpoint inhibitors, monoclonal antibodies and chimeric antigen receptor T cells—both in preclinical mouse models and in clinical trials. Combination therapies have the advantage of targeting multiple mechanisms to enhance the immune response against tumors (for reviews see 8 49 50). Combination of NIZ985 with spartalizumab has been initiated in Japan in patients with advanced solid tumors and lymphoma (NCT04261439). Additionally, several phase I trials include the use of IL-15 with nivolumab (anti-PD-1) and ipilimumab (anti-cytotoxic T-lymphocyte associated protein 4 [CTLA4]) in refractory cancers (NCT03388632), with avelumab (anti-PD-L1) in T cell malignancies (NCT03905135), with obinutuzumab (anti-CD20) in chronic lymphocytic leukemia (NCT03759184), and with alemtuzumab (anti-CD52) in adult T-cell leukemia (NCT02689453). The results from the phase I trial of the heterodimeric molecule N-803 delivered subcutaneously in combination with nivolumab in patients with metastatic non-small cell lung cancer have been recently reported.^51^ Another promising combination strategy is the use of N-803 together with bi/tri-specific antibodies (BiKE and TriKE). BiKE and TriKE consist of the single-chain variable fragments against both NK activating receptors and tumor associated antigen(s), such as CD19, CD20 and CD33, linked to human IL-15.^52 53^

In conclusion, these promising first-in-human data point to NIZ985 as an immunotherapeutic suitable for further clinical evaluation to optimize its dosing schedule and use in combination regimens, in order to leverage fully the potentially favorable immunologic changes observed during treatment.

## Data Availability

No data are available.
