# Supplementary material for: Phase I study of single agent NIZ985, a recombinant heterodimeric IL-15 agonist, in adult patients with metastatic or unresectable solid tumors
Source: J Immunother Cancer. 2021 Nov 19;9(11):e003388. doi: 10.1136/jitc-2021-003388 (PMC8606766; doi:10.1136/jitc-2021-003388)
Supplement: Supplementary data [file jitc-2021-003388supp001.pdf]

Supplementary Data

Table S1. Novartis flow cytometry methods and panel details. 2

Table S2. NCI flow cytometry panel details. 3

Table S3. Prior treatment and disease diagnosis. 4

Table S4. Patient outcomes. 5

Table S5. Summary of adverse events (all grades) irrespective of relationship to study treatment. 6

Table S6. Proportion of lymphocyte subsets expressing Ki67 (Peak fold-change during treatment over baseline) 7

Figure S1. Duration of treatment 8

Figure S2. Injection site reactions. 9

Figure S3: Best percentage change from baseline in sum of diameters of target lesions. 10

Figure S4. Individual proportions of $Ki67^+$ $CD4^+$ cells, $CD8^+$ cells, γδ T cells and NK cells over the first 2-3 treatment cycles among the last five patients treated in the study. 11

Figure S5. Absolute count of blood lymphocyte subsets after treatment with NIZ985. 12

Conlon K, *et al. J Immunother Cancer* 2021; 9:e003388. doi: 10.1136/jitc-2021-003388

Table S1. Novartis flow cytometry methods and panel details.

The following panel and methodology were used to assess the last five patients treated, following study expansion to a multicenter trial.

Fluorochrome	Marker	Manufacturer	Catalog #	Clone
**BUV395**	CD45	BD	563792	HI30
**BUV737**	CD4	BD	612748	SK3
**BV421**	CD8	BD	562428	RPA-T8
**eF506**	Viability	ThermoFisher	65-0866-14	N/A
**BV650**	TIM-3	BD	565564	7D3
	CD25	BD	563719	M-A251
**BV711**	CD45RA	BD	563733	HI100
	CD335	BD	563043	NKp46
**BV786**	HLA-DR	BD	564041	G46-6
**FITC**	CD3	ThermoFisher	11-0036-42	SK7
	CD159a	Miltenyi Biotec	130-113-565	REA110
**PerCP-Cy5.5**	TCR Vδ2	Biolegend	331424	B6
	CD3	ThermoFisher	45-0036-42	SK7
**PE**	CCR7	BD	560765	150503
	CD56	BD	340363	NCAM16.2
**PE-eF610/ PE-CF594**	LAG-3	ThermoFisher	61-2239-42	3DS223H
	NKG2D	BD	562498	1D11
**PE-Cy7**	PD-1	BD	561272	EH12.1
	CD14	Biolegend	325618	HCD14
**Alexa Fluor 647**	Ki-67	BD	558615	B56
**APC-eF780**	CD16	ThermoFisher	47-0168-42	eBioCB16
	CD38	ThermoFisher	47-0389-42	HIT2
**Methodology**	• Briefly, up to $4x10^6$ cryopreserved PBMCs were thawed quickly in a 37ºC water bath and washed by centrifugation with pre-warmed RPMI medium containing 10% FBS. After washing, cells were stained with fixable viability dye eFluor™506 (ThermoFisher, San Diego, CA) at a dilution of 1:100 in wash buffer (phosphate-buffered saline containing 0.1% sodium azide and 2% fetal bovine serum) followed by staining with fluorochrome-conjugated surface antibodies for 30 minutes at room temperature in the dark. Previously prepared and qualified antibody cocktails consisting of 12 surface antibodies were utilized.<br>• After incubation, cells were washed once by centrifugation in wash buffer and fixed with 1x Fixation/Permeabilization Buffer from the Foxp3/Transcription Factor Staining Buffer Set (ThermoFisher, San Diego, CA) for 30 minutes at room temperature in the dark. After fixation, cells were washed once by centrifugation with 1x Permeabilization Buffer from the Foxp3/Transcription Factor Staining Buffer Set and resuspended in 1x Permeabilization Buffer. Fluorochrome-conjugated intracellular antibody (Ki-67) was added and cells were incubated for 30 minutes at room temperature in the dark. Cells were then washed twice by centrifugation with 1x Permeabilization Buffer and resuspended in 0.5% Formalin solution.<br>• Cells were then acquired on a BD LSRFortessa X-20 equipped with 5 lasers (BD Biosciences, San Jose, CA) and data was analyzed using FlowJo software (FlowJo LLC, Ashland, OR). For data analysis of the T-cell-Proliferation assay, after exclusion of debris, doublets and dead cells, T cells were further gated for CD4+ and CD8+ subsets. Both CD4+ and CD8+ T cells were then subsequently analyzed for the expression of various biomarkers to define proliferating (Ki-67), checkpoint inhibitor-expressing (PD-1, LAG-3, TIM-3), activated (CD38, HLA-DR) and memory (CCR7, CD45RA) T cell subsets. For data analysis of the NK-cell-Proliferation assay, after exclusion of debris, doublets and dead cells, NK cells were further gated for NK subsets (CD56, CD16), as well as inhibition (CD159a) and activation (CD25, CD335, NKG2D) biomarkers. Monocytes were also assessed for CD14, CD16 and HLA-DR expression.			

Table S2. NCI flow cytometry panel details.

The following monoclonal antibodies were used for the flow analyses in the first nine patients treated in the study: from BD Biosciences: CXCR5-BUV395 (clone RF8B2), CD16-BUV496 (clone 3G8), CD14-BUV805 (clone M5E2), FoxP3-BV450 (clone 236A/E7), CD28-BV480 (clone L293), CD4-BV750 (clone SK3), CD45RA-BB700 (clone 5H9), CD279-PECF594 (clone EH12.1), CD152-PECy5 (clone BNI3), CD25-PECy7 (clone M-A251), Ki-67-Ax647 (clone B56), Granzyme B-Ax700 (clone GB11), CD20-BUV395 (clone 2H7), CD56-BV510 (clone NCAM16.2), CD94-BV786 (clone HP-3D9) and CD3 custom conjugated with BB660 (cloneSP34-2). From BioLegend: CD8-BV570 (clone RPA-T8), CD127-BV605 (clone A019D5), CCR6-BV650 (clone G034E2), Vd2-BV711 (clone B6), CCR7-BV785 (clone G043H7), Granzyme A-PE (clone CB9), CXCR3-APCCy7 (clone G025H7), CD80-BV421 (clone 2D10), HLA-DR-BV570 (clone L243), CD303-BV605 (clone 201A), CD11c-BV650 (clone 3.9) and CD86-BV711 (clone IT2.2). From ThermoFisher: Vd1-FITC (clone TS8.2). From Beckman Coulter: NKG2a-PECy7 (clone Z199). The CD11b antibody (clone ICRF44) was conjugated in-house with FITC. The viability dye (LIVE/DEAD fixable Blue dead cell stain) was from Invitrogen. Two different staining cocktails were used (T cell panel, NK and myeloid panel) as indicated below.

T cell panel

Detector	Marker	Clone	Fluor	Source
UV395	CXCR5	RF8B2	BUV395	BD
UV450	LIVE/DEAD fixable Blue Dead Cell Stain			Invitrogen
UV500	CD16	3G8	BUV496	BD
UV795	CD14	M5E2	BUV805	BD
V440	***FoxP3***	***236A/E7***	***BV450***	***BD***
V510	CD28	L293	BV480	BD
V570	CD8	RPA-T8	BV570	BioLegend
V605	CD127	A019D5	BV605	BioLegend
V650	CCR6	G034E2	BV650	BioLegend
V710	V delta 2	B6	BV711	BioLegend
V750	CD4	SK3	BV750	BD
V800	CCR7	G043H7	BV785	BioLegend
B515	V delta 1	TS8.2	FITC	Thermo
B660	CD3	SP34-2	BB660	Custom (BD)
B710	CD45RA	5H9	BB700	BD
G575	***Gran A***	***CB9***	***PE***	***Biolegend***
G610	CD279/PD-1	EH12.1	CF594PE	BD
G660	CD152	BNI3	Cy5PE	BD
G780	CD25	M-A25I	Cy7-PE	BD
R660	***Ki-67***	***B56***	***Ax647***	***BD***
R710	***Granzyme B***	***GB11***	***Ax700***	***BD***
R780	CXCR3	G025H7	Cy7APC	BioLegend

NK and myeloid panel

Detector	Marker	Clone	Fluor	Source
UV395	CD20	2H7	BUV395	BD
UV450	LIVE/DEAD fixable Blue Dead Cell Stain			Invitrogen
UV500	CD16	3G8	BUV496	BD
UV795	CD14	M5E2	BUV805	BD
V440	CD80	2D10	BV421	Biolegend
V510	CD56	NCAM16.2	BV510	BD
V570	HLA-DR	L243	BV570	Biolegend
V605	CD303	201A	BV605	Biolegend
V650	CD11c	3.9	BV650	Biolegend
V710	CD86	IT2.2	BV711	Biolegend
V750	CD4	SK3	BV750	BD
V800	CD94	HP-3D9	BV786	BD
B515	CD11b	ICRF44	FITC	VRC conjugate
B660	CD3	SP34-2	BB660	Custom (BD)
G575	***Gran A***	***CB9***	***PE***	***Biolegend***
G610	CD279/PD-1	EH12.1	CF594PE	BD
G780	NKG2a	Z199	Cy7PE	Beckman Coulter
R660	***Ki-67***	***B56***	***Ax647***	***BD***
R710	***Granzyme B***	***GB11***	***Ax700***	***BD***

Intracellular antibodies are bold and italicized

Conlon K, *et al. J Immunother Cancer* 2021; 9:e003388. doi: 10.1136/jitc-2021-003388

Table S3. Prior treatment and disease diagnosis.

Characteristic	NIZ985 0.25 µg/kg n=1	NIZ985 0.5 µg/kg n=2	NIZ985 1.0 µg/kg n=6	NIZ985 2.0 µg/kg n=3	NIZ985 4.0 µg/kg n=2	All NIZ985 patients N=14
**Number of prior antineoplastic regimes, n (%)**						
1	0	0	1 (16.7)	2 (66.7)	0	3 (21.4)
2	0	1 (50.0)	1 (16.7)	1 (33.3)	1 (50.0)	4 (28.6)
≥3	1 (100)	1 (50.0)	4 (66.7)	0	1 (50.0)	7 (50.0)
**Prior medication, n (%)**						
Chemotherapy	1 (100)	1 (50.0)	4 (66.7)	3 (100)	1 (50.0)	10 (71.4)
Hormonal therapy	0	1 (50.0)	0	0	0	1 (7.1)
Immunotherapy	0	2 (100)	3 (50.0)	2 (66.7)	2 (100)	9 (64.3)
Anti-CTLA4	0	1 (50.0)	2 (33.3)	0	0	3 (21.4)
Anti-PD-1	0	2 (100)	3 (50.0)	0	1 (50.0)	6 (42.9)
Targeted therapy	0	0	0	0	1 (50.0)	1 (7.1)
Vaccine	0	0	1(16.7)	0	0	1 (7.1)
Other	0	0	3 (50.0)	0	0	3 (21.4)
**Disease diagnosis, n (%)**						
Breast cancer	0	0	1 (16.7)	0	0	1 (7.1)
Cholangiocarcinoma	0	0	0	1 (33.3)	0	1 (7.1)
Colorectal cancer	0	0	1 (16.7)	1 (33.3)	0	2 (14.3)
Cutaneous melanoma	0	1 (50.0)	1 (16.7)	0	0	2 (14.3)
Metastatic renal cell carcinoma	1 (100)	0	0	0	0	1 (7.1)
Neuroendocrine	0	0	0	0	1 (50.0)	1 (7.1)
Renal carcinoma	0	0	0	0	1 (50.0)	1 (7.1)
Thyroid cancer	0	0	1 (16.7)	0	0	1 (7.1)
Uveal melanoma	0	0	1 (16.7)	0	0	1 (7.1)
Leiomyosarcoma	0	1 (50.0)	0	0	0	1 (7.1)
Osteosarcoma	0	0	1 (16.7)	0	0	1 (7.1)
Carcinoma ex pleomorphic adenoma	0	0	0	1 (33.3)	0	1 (7.1)

CTLA4, cytotoxic T-lymphocyte-associated protein 4; PD-1, programmed cell death-1.

Table S4. Patient outcomes.

	NIZ985 0.25 µg/kg n=1	NIZ985 0.5 µg/kg n=2	NIZ985 1.0 µg/kg n=6	NIZ985 2.0 µg/kg n=3	NIZ985 4.0 µg/kg n=2	All NIZ985 patients N=14
**Treatment ongoing, n (%)**	0	0	1 (16.7)	0	0	1 (7.1)
**End of treatment, n (%)**	1 (100)	2 (100)	5 (83.3)	3 (100)	2 (100)	13 (92.9)
Disease progression	1 (100)	1 (50.0)	4 (66.7)	2 (66.7)	0	8 (57.1)
Adverse event	0	1 (50.0)	0	1 (33.3)	1 (50.0)	3 (21.4)
Physician decision	0	0	0	0	1 (50.0)	1 (7.1)
Participant withdrawal	0	0	1 (16.7)	0	0	1 (7.1)

Table S5. Summary of adverse events (all grades) irrespective of relationship to study treatment.

Adverse event, n (%)	NIZ985 0.25 µg/kg n=1	NIZ985 0.5 µg/kg n=2	NIZ98 1.0 µg/kg n=6	NIZ985 2.0 µg/kg n=3	NIZ985 4.0 µg/kg n=2	All NIZ985 patients N=14
Any event<br>Grade 3–4	1 (100)<br>0	2 (100)<br>1 (50.0)*	6 (100)<br>2 (33.3)†	3 (100)<br>1 (33.3)§	2 (100)<br>2 (100)‡	14 (100)<br>6 (42.9)
**Common adverse events (>20% in all patients)**						
ISR	1 (100)	2 (100)	6 (100)	3 (100)	2 (100)	14 (100)
Chills	0	1 (50.0)	4 (66.7)	3 (100)	2 (100)	10 (71.4)
Fatigue	0	1 (50.0)	4 (66.7)	3 (100)	1 (50.0)	9 (64.3)
Pyrexia	0	1 (50.0)	4 (66.7)	2 (66.7)	0	7 (50.0)
Constipation	0	0	2 (33.3)	2 (66.7)	1 (50.0)	5 (35.7)
Nausea	0	1 (50.0)	2 (33.3)	1 (33.3)	1 (50.0)	5 (35.7)
Arthralgia	0	2 (100)	2 (33.3)	1 (33.3)	0	5 (35.7)
Decreased appetite	0	0	2 (33.3)	1 (33.3)	1 (50.0)	4 (28.6)
Hyperhidrosis	0	0	2 (33.3)	2 (66.7)	0	4 (28.6)
Anemia	0	1 (50.0)	1 (16.7)	1 (33.3)	0	3 (21.4)
Diarrhea	1 (100)	0	1 (16.7)	0	1 (50.0)	3 (21.4)
Vomiting	0	0	2 (33.3)	1 (33.3)	0	3 (21.4)
Back pain	1 (100)	1 (50.0)	0	0	1 (50.0)	3 (21.4)
Myalgia	0	1 (50.0)	2 (33.3)	0	0	3 (21.4)
Cognitive disorder	0	1 (50.0)	1 (16.7)	0	1 (50.0)	3 (21.4)
Dyspnea	0	0	1 (16.7)	1 (33.3)	1 (50.0)	3 (21.4)
Hypotension	0	0	0	2 (66.7)	1 (50.0)	3 (21.4)
Pruritus	0	0	1 (16.7)	1 (33.3)	1 (50.0)	3 (21.4)

*Grade 3 events of anemia, small intestinal obstruction, and cellulitis.

†Grade 3 events of ISR (subsequently reclassified as grade 2 on review), hyperglycemia (3 events), non-cardiac chest pain, and increased international normalized ratio in one patient, and grade 3 diarrhea in one patient.

§Grade 3 increase in international normalized ratio.

‡Grade 3 hyponatremia in one patient; grade 3 and/or 4 events of lymphocyte count decreased (2 events), productive cough, dysphonia, dysphagia, stridor, bronchial obstruction, tracheal stenosis, esophageal stenosis, esophageal pain, decreased appetite, dehydration, hypotension, embolism (2 events), atrial fibrillation, fatigue, peripheral edema, purpura, acute kidney injury, oliguria and vasculitis (2 events) in one patient.

ISR, injection site reaction.

Table S6. Proportion of lymphocyte subsets expressing Ki67 (Peak fold-change during treatment over baseline)

Dose	NK cells	CD8⁺ T cells	CD4⁺ T cells	γδ T cells
**1 μg/kg** (n=6)	4.9	4.4	2.1	7.5
	11.5	5.5	3.2	29.7
	8.2	2.6	5.1	5.1
	n.a.	3	3.0	n.a.
	8.2	5	2.5	17
	n.a.	3.8	4.7	79.3
**2 μg/kg** (n=3)	6.3	5.2	1.2	4.1
	5.9	5.4	5	4.1
	10.7	11	5.4	15.1
**4 μg/kg** (n=1)	2.1	2.0	0.9	3.5

Note: For each patient, fold-change induction of Ki67 expressing cells was determined at multiple timepoints during Cycle 1 by comparing to pre-treatment baseline value. The maximum observed increase in Ki67 expression for each patient is reported above. n.a. = not available.

Figure S1. Duration of treatment

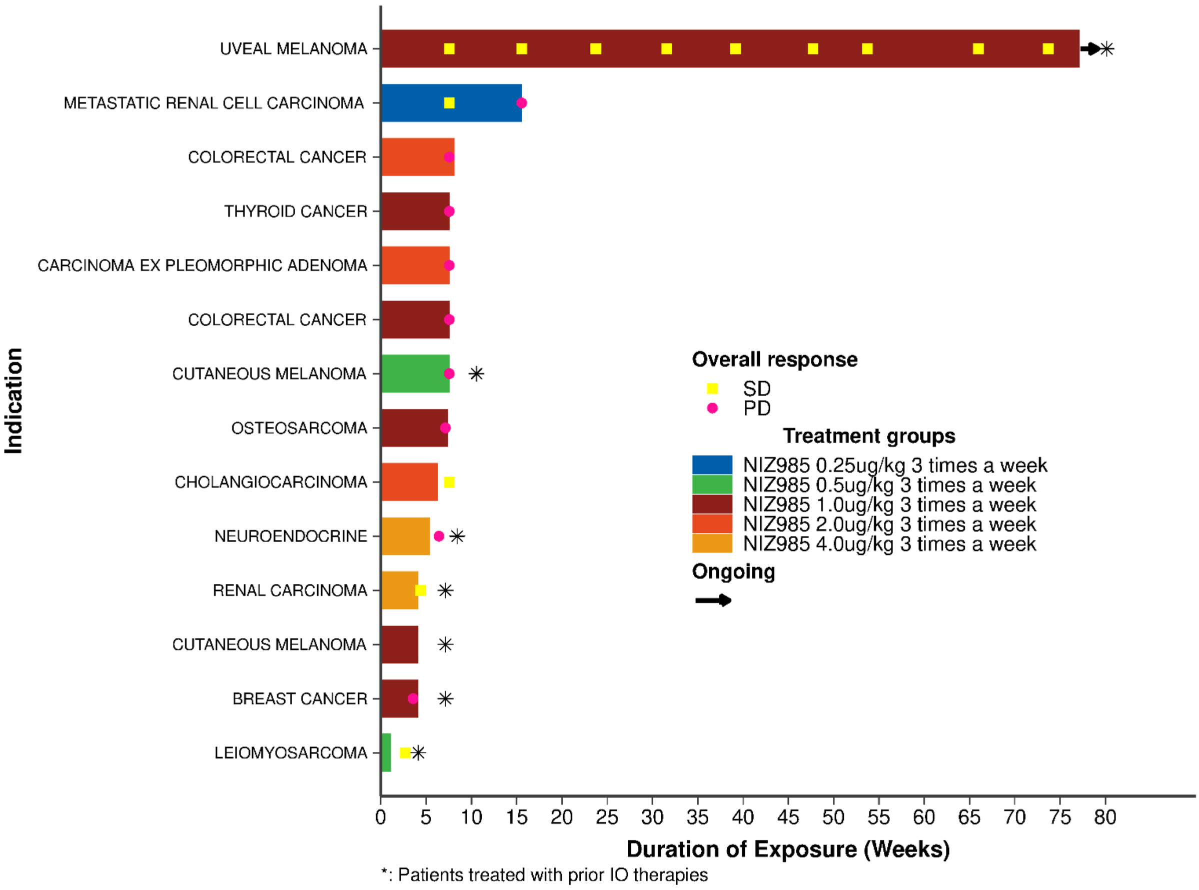

IO, immuno-oncology; PD, progressive disease; SD, stable disease.

Figure S2. Injection site reactions.

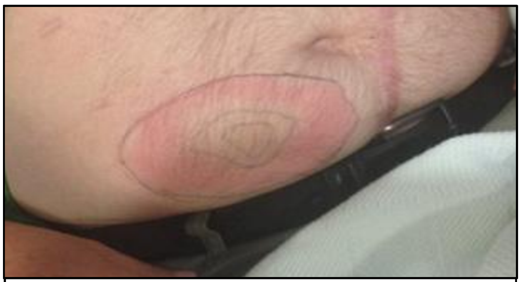

0.25 µg/kg patient Cycle 1, Day 1 injection site on Cycle 1, Day 5

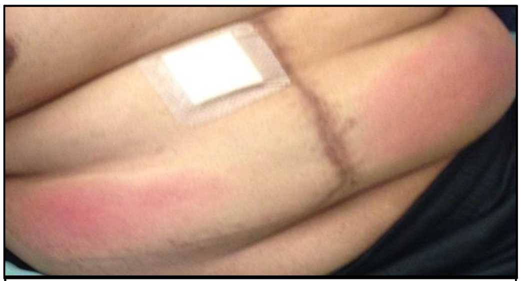

1 µg/kg patient Cycle 1, Day 3 and 5 injection sites on Cycle 1, Day 10

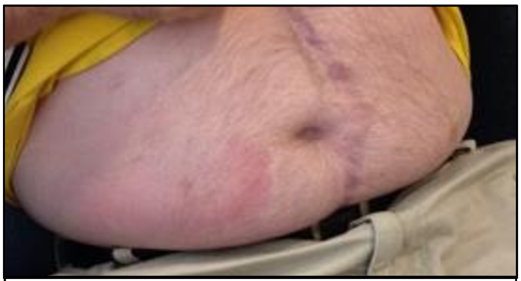

0.25 µg/kg patient Cycle 3, Day 1 injection site on Cycle 3, Day 8

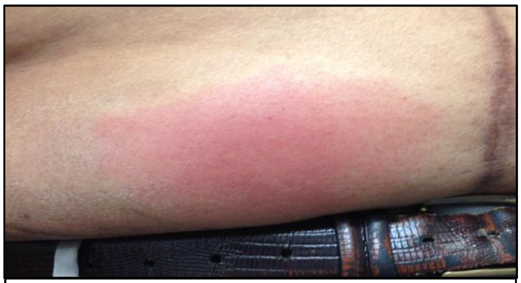

1 µg/kg patient Cycle 2, Day 5 injection site on Cycle 2, Day 8

Figure S3: Best percentage change from baseline in sum of diameters of target lesions.

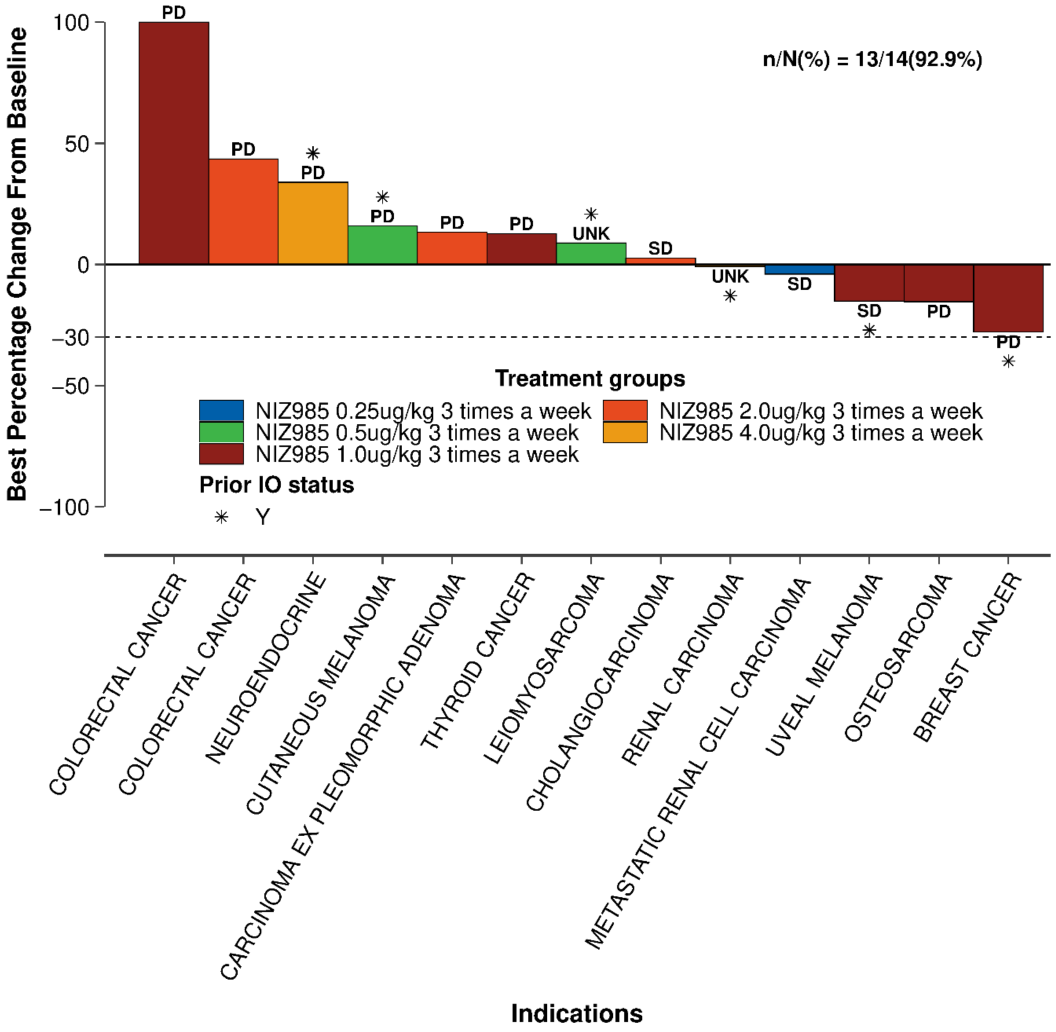

IO, immuno-oncology treatment; PD, progressive disease; SD, stable disease; UNK, unknown. One patient (1.0 µg/kg) had no recorded data and is excluded from the plot.

Figure S4. Individual proportions of Ki67^{+} CD4^{+} cells, CD8^{+} cells, γδ T cells and NK cells over the first 2-3 treatment cycles among the last five patients treated in the study. (see **table S1** for methodology).

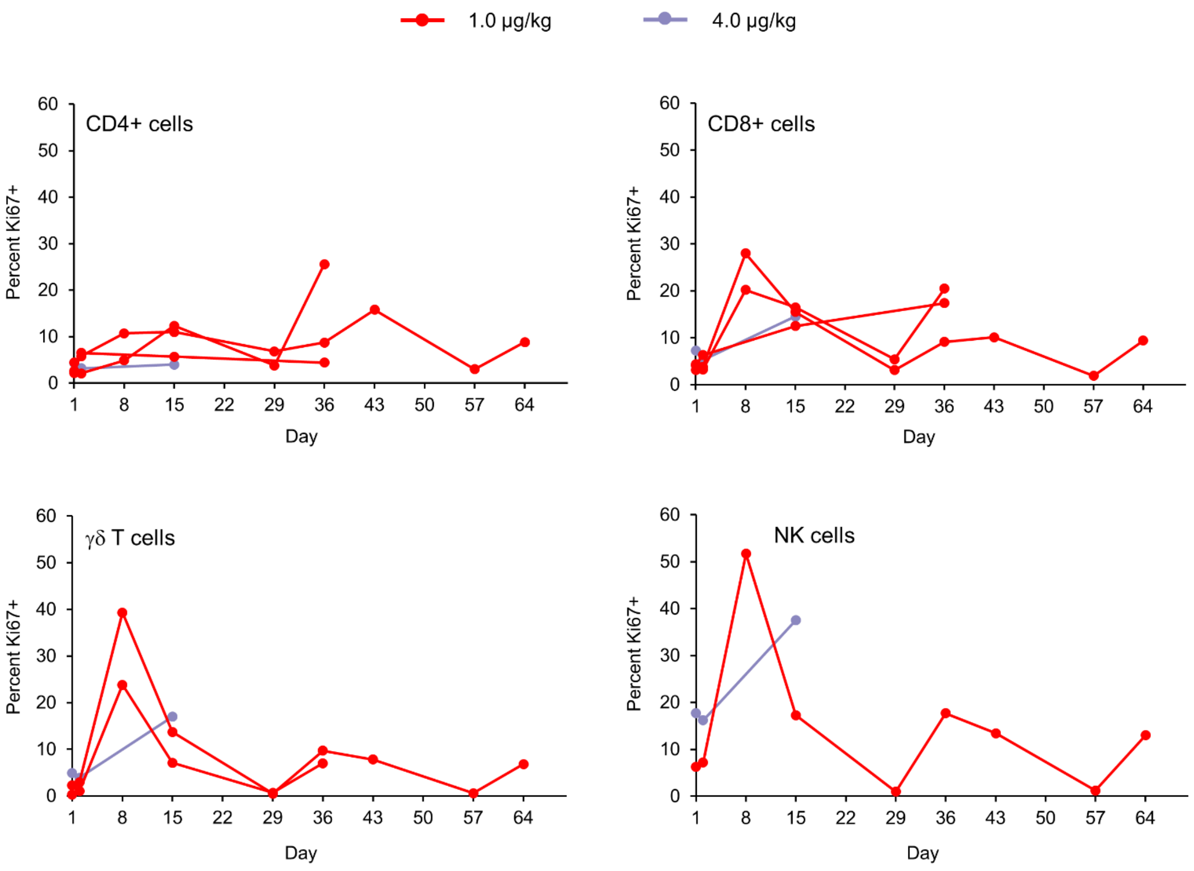

NK, natural killer.

Figure S5. Absolute count of blood lymphocyte subsets after treatment with NIZ985. Peripheral blood samples were obtained during Cycle 1 of treatment with NIZ985 and analyzed by flow cytometry. Plotted results represent available absolute cell counts for individual patients, normalized to Cycle 1 Day 1 of each patient. Gray bars represent the period of NIZ985 administration.

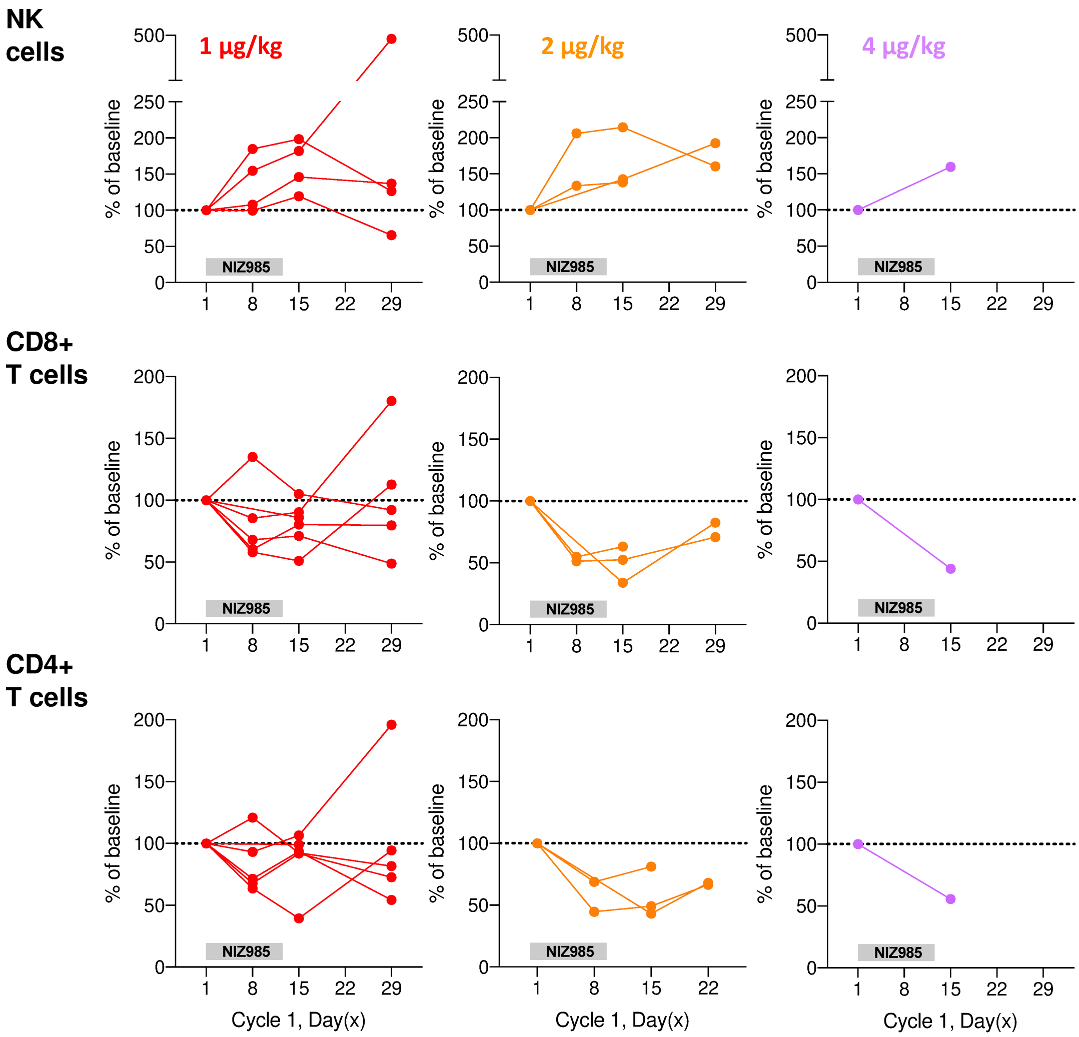

Conlon K, et al. J Immunother Cancer 2021; 9:e003388. doi: 10.1136/jitc-2021-003388
